# Advancing translational science through trial integrity: REDCap-based approaches to mitigating fraud and bias

**DOI:** 10.1017/cts.2025.10176

**Published:** 2025-10-24

**Authors:** Gaylen E. Fronk, Larry W. Hawk, Andrew Cates, John Clark, Noelle Natale, Jennifer Dahne

**Affiliations:** 1 Department of Psychiatry and Behavioral Sciences, Medical University of South Carolina, Charleston, SC, USA; 2 Department of Psychology, University at Buffalo, Buffalo, NY, USA; 3 Information Solutions, Medical University of South Carolina, Charleston, SC, USA; 4 Hollings Cancer Center, Medical University of South Carolina, Charleston, SC, USA

**Keywords:** Decentralized clinical trials, redcap, fraud, bias, digital tools

## Abstract

Decentralized clinical trials (DCTs) have the potential to increase pace and reach of recruitment as well as to improve sample representation, compared to traditional in-person clinical trials. However, concerns linger regarding data integrity in DCTs due to threats of fraud and sampling bias. The purpose of this report is to describe two tools that we have developed and successfully implemented to combat these threats. Cheatblocker and QuotaConfig are two external modules that we have made publicly available within the REDCap data capture system to target fraud and sampling bias, respectively. We describe the modules, present two case examples in which we used the modules successfully, and discuss the potential impact of tools such as these on data integrity in DCTs. We situate this discussion within the broader landscape of translational science wherein we strive to improve research rigor and efficiency to maximize public health benefit.

Decentralized clinical trials (DCTs), also called remote or virtual trials, offer a promising alternative to traditional clinical trials [1,2]. Traditional clinical trials require in-person participation and typically take place at large, urban academic medical centers. Unfortunately, these factors have produced samples that often do not represent the real-world clinical populations these trials aim to study and help [3]. For example, traditional clinical trials focused on mental health disorders tend to have younger participants with lower disease severity, fewer co-occurring conditions, and higher socioeconomic status than corresponding real-world populations [3]. In other words, individuals who face the highest impact of a disease are not the same individuals who participate in research.

DCTs have the potential to recruit larger, more representative samples [4,5]. They rely on technology and digital tools to permit cost-effective participation from a broader geographic area without in-person requirements. These methods enable increased participation from individuals from rural areas (i.e., farther from large research institutions); those who face time or access barriers (e.g., individuals working third shifts, individuals who cannot afford childcare or time off work to attend in-person visits); and those whose disease severity prevents in-person participation [6–8]. Patients also prefer participating in DCTs compared to traditional clinical trials with in-person requirements, citing reduced burden (e.g., less time taken up by in-person clinic visits) and benefits of technology-based data collection (e.g., believed data collection would be more accurate and objective, possibility of tracking their own health, using interesting technology) [9]. Altogether, DCTs are well-positioned to address long-standing issues in traditional clinical research and to improve generalizability to people disproportionately affected by the diseases we study.

Despite clear promise of DCT designs, concerns linger related to data integrity due to the risk of fraud [10,11]. Fraud involves providing false data, including misrepresenting trial eligibility criteria or study outcomes. Fraud can be intentional or unintentional, and there are many reasons why a potential study participant might provide fraudulent responses. Although fraud is not unique to DCTs, risk may be greater without in-person participation. For example, a recent review documents 7 DCTs where fraud caused consequences as severe as suspending trials [12], and misrepresenting data is a known issue in online research using platforms such as mTurk [13,14].

Fraud can have far-reaching, negative impacts for research and public health [12,14]. For example, a trial may conclude erroneously that a treatment is effective based on incorrect participant reports of outcomes. Similarly, a treatment may be “destined to succeed” if individuals without the targeted condition participate; at follow-up, these individuals may report remission when in fact they never had symptoms [15]. In either case, an ineffective treatment will appear effective and be disseminated to patients, which negatively impacts public health by failing to reduce disease prevalence and associated societal costs.

Some people intentionally perpetrate fraud in research studies, often solely for remuneration. One common form of intentional fraud is providing false screening data to gain entry to a research study. These individuals may repeatedly change demographic or health-related characteristics when completing screening forms to guess eligibility criteria. This tactic can affect not only study outcomes and subsequent public health outcomes but also sample representativeness. If individuals can misrepresent key inclusion criteria, sample composition cannot be trusted.

Beyond the risk from intentional fraud, including misrepresentation during self-screening, sampling bias may further compromise data integrity. Concerns remain as to whether study samples in DCTs will actually be more representative than samples in traditional trials [16]. DCTs can increase recruitment pace and reach, but they are still subject to sampling bias. Individuals with lower household incomes or who are from rural communities may experience limited or less stable internet access and subsequently may participate less in online research [17]. Women, White adults, and younger individuals may be more likely to respond to online advertisements [18–20]. Consequently, DCTs run the risk of perpetuating existing disparities in clinical research and subsequent public health care.

To ensure DCTs live up to their promise, we need tools that can address issues of data integrity related to fraud and sample representativeness. These tools must align with DCT methods such that they are both available for remote deployment and sufficiently automated to meet a faster recruitment pace. Moreover, these tools should integrate into existing infrastructure that can be disseminated to multiple investigators or research sites so all can benefit.

The purpose of this report is to address a translational science question: How can we improve data integrity in DCTs? We describe two tools that may offer solutions to counter threats to data integrity from fraud and sampling bias. Our research group has developed two REDCap external modules: CheatBlocker, to address fraud from misrepresentation during self-screening, and QuotaConfig, to address sample representation. We describe the modules and discuss two DCT case examples in which we have successfully implemented these modules. Finally, we situate these modules within the broader translational science landscape of how we can leverage digital tools to improve data integrity within DCT research.

## Tool development

We developed our tools in REDCap, a web-based research data capture system [21,22]. REDCap is freely available to research institutions and currently boasts 3.6 million users across 7,730 institutions in 160 countries. This system permits data collection via customizable surveys and data storage within a project’s database.

In addition to individual project databases, REDCap offers a centralized repository of External Modules – add-on packages of software that extend and customize functionality at the project or institution level. Software developers create and submit modules, and REDCap administrators download these modules for use within their institution. Consequently, REDCap offers a platform to integrate shared tools into existing project infrastructures and disseminate them widely.

We developed two external modules and shared them on the REDCap repository of external modules, thus making them publicly available. Any researchers using REDCap can download these modules for integration within their own projects. These modules can be used independently or together.

### CheatBlocker

The REDCap external module “Cheatblocker” addresses the risk of fraud – both intentional and unintentional – to prevent individuals from completing an online study screener multiple times to gain study entrance. This module coordinates with the project screening survey to identify potential duplicate (and thereby fraudulent) records using a customizable set of screening questions. To avoid biasing responses, individuals completing a screening survey do not know that the module is running in the background.

**Figure 1.**
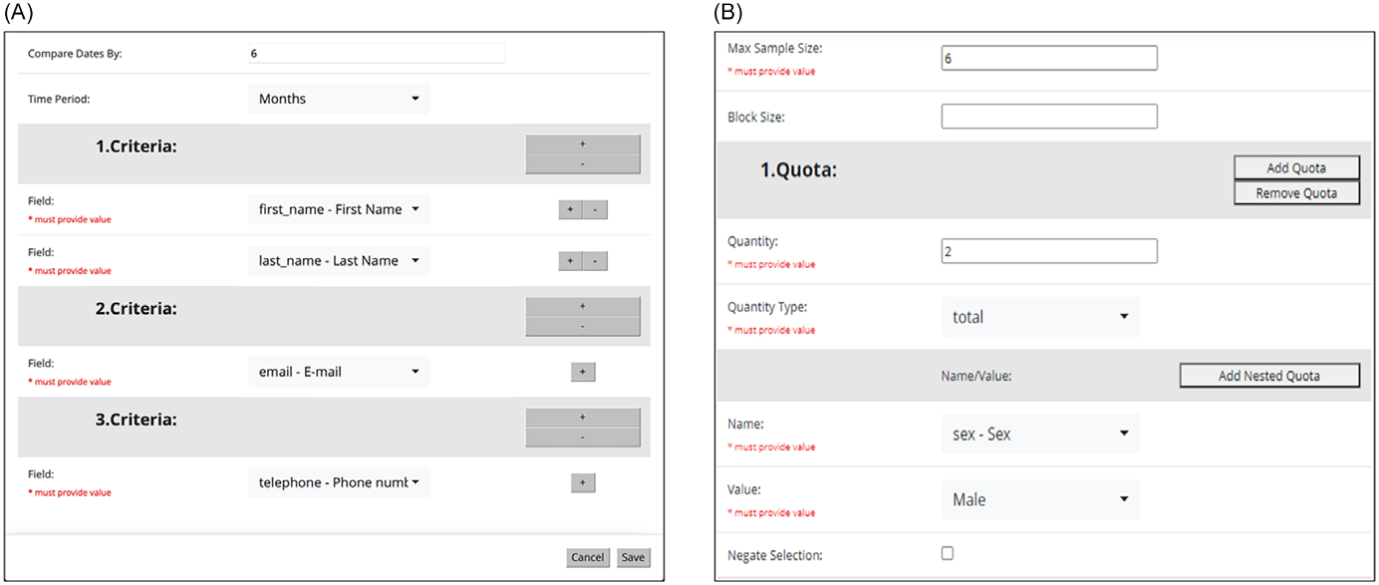
Cheatblocker and QuotaConfig examples. **Panel A**. Screenshot example from Cheatblocker module in REDCap. Researchers can set the time period within which to compare dates. They then select the criteria for flagging duplicates. In this example, the researcher has selected to compare records submitted within 6 months and to check for the same first and last name, or the same email address, or the same phone number. Fields *within* a “Criteria” section indicate “AND” logic (e.g., first AND last name). Fields *across* “Criteria” sections indicate “OR” logic (e.g., email OR phone number). **Panel B**. Screenshot example from QuotaConfig in REDCap. Researchers enter their maximum sample size and, optionally, a block size to monitor enrollment in blocks rather than across the full sample. They then select enrollment minimums or maximums. In this example, the researcher has set the full sample size to 6, has decided not to use blocks, and has set the quota that no more than 2 male participants (of 6 total) may be enrolled.

To implement Cheatblocker, researchers select fields from their screening survey (e.g., identifiers like name, date of birth, phone number; study-specific eligibility criteria) to use for fraud detection. Then, they add the desired cheat-blocking criteria to their project to determine which fields or combinations of fields should indicate potential fraud. For example, identical name and date of birth OR identical name and email address across screening survey submissions completed at different times could flag these records as potential duplicates. Investigators have complete flexibility over which fields from the screening survey Cheatblocker will use to determine if records are duplicative; no other fields in the screening survey will be checked by the module. This process allows researchers to customize criteria to be study-relevant (e.g., Figure 1A).

Additionally, researchers can customize the date range for the duplicate check. For example, a project could stipulate that only duplicate records submitted within 1 year of one another would be flagged. This feature may help differentiate intentional fraud from mistakes or newly eligible participants. Duplicate entries submitted in rapid succession likely represent intentional fraud (Table 1). Duplicate entries submitted a year apart may be genuine mistakes, or they may represent participants whose eligibility should be reconsidered (e.g., health behaviors or clinical status have changed, they have moved, or they are now old enough to qualify). In these cases, study staff could reach out to reevaluate eligibility.

**Table 1.** Cheatblocker results across case studies

	COast project	VapeX project
Total screenings completed	4,844	2,845
Original duplicates^a^ (N, %)	526 (10.86%)	175 (6.15%)
Duplicate entries^b^ (N, %)	971 (20.05%)	538 (18.91%)
Total duplicates (N, %)	1,497 (30.90%)	713 (25.06%)
Range (duplicates per original)	1–13	1–15
**Duplicated fields**		
Three (of 3) fields duplicated		
*Full name + email + phone number (N)*	781	379
Two (of 3) fields duplicated		
*Email + phone number (N)*	269	119
*Full name + email (N)*	78	21
*Full name + phone number (N)*	63	21
One (of 3) field duplicated		
*Phone number (N)*	103	118
*Email (N)*	47	32
*Full name (N)*	32	15
**Time between submissions among duplicates**		
Same day (%)	28.32%	71.33%
1 – 3 days (%)	8.11%	6.76%
4 – 7 days (%)	6.91%	3.96%
8 – 14 days (%)	7.71%	3.73%
2 weeks – 1 month (%)	20.35%	3.96%
Greater than 1 month (%)	28.59%	10.26%

^a^Original duplicates: The original record duplicated in a subsequent record.

^b^Duplicate entries: Subsequent records that matched one or more previous submissions.

N = number (of participants).

Researchers can also gain insight into why submissions are flagged as duplicates. For any record flagged, the specific failed criteria will automatically populate into the screening survey record in REDCap for staff review (Table 1). This feature permits identifying which fields most commonly trigger the flag and, conversely, which fields individuals perpetrating intentional fraud know to change. For example, entries may be frequently flagged for the same phone number, but across these entries with the same phone number, potential participants know to change their names and email addresses. Researchers could pool data within their lab, explore differences across populations (e.g., whether older and younger participants fail for different reasons), and guide future research.

Different patterns of responding may also elucidate intention to allow study staff to distinguish intentional fraud from genuine mistakes. If duplicate entries have all the same contact information (e.g., top row in Table 1 under “Duplicated fields”), there was no attempt to disguise identity, suggesting these submissions may have come from individuals who accidentally completed the same screening survey twice. If duplicate entries have only some fields changed (e.g., different name but same email address and phone number), this may suggest intentional fraud.

Finally, researchers implementing this module can decide whether to inform potential participants that they are ineligible automatically at the point of screening or to flag a record as a potential duplicate and allow study staff to make a final decision regarding record inclusion. This decision requires weighing staff burden against anticipated recruiting success. Automatically informing potential participants they are ineligible eliminates staff time, which can be considerable in web-based research that boasts high rate and pace of recruitment. However, a fully automated process may conservatively eliminate participants who are in fact eligible. This approach may be costly if the project targets a difficult-to-reach population or one characterized by a rare trait; additional staff time may be worth ensuring no eligible participants will be excluded. In these cases, using Cheatblocker’s partially automated alternative, in which staff review only records flagged as potential duplicates, may be preferred. Other Cheatblocker features can support staff confidence when manually reviewing eligibility. For example, if multiple records are submitted close together in time, with the same phone number and email address but different names, they are likely fraudulent.

Cheatblocker provides a generalizable template while offering customizability to individual researchers regarding which fields to use and how to flag records. To date, this module has been used in hundreds of projects across 39 institutions. Detailed instructions for using the Cheatblocker module are publicly available (https://github.com/MUSC-BMIC/redcap_cheat_blocker).

### QuotaConfig

The REDCap external module “QuotaConfig” addresses risk of sampling bias at study screening. This module coordinates with the project screening survey to categorize potential participants according to project-specific sampling needs. To avoid biasing responses, individuals completing a screening survey do not know the module is running in the background.

To implement QuotaConfig, researchers select characteristics (e.g., sex, race/ethnicity, age, rurality, disease severity) from their screening survey that are critical for study sample representativeness. For these variables, researchers can specify enrollment minimums or maximums using quantity (i.e., number of participants) or percentage of the total sample size (Figure 1B). For example, a research team could set an enrollment minimum for women participants at 25% of the sample. Once 75% of available spots in the study are filled by non-women participants, only women participants will be permitted study entry. Researchers may set multiple enrollment criteria (e.g., minimums for women and rural participants) as well as “nested” criteria that consider multiple characteristics simultaneously (e.g., minimums for rural women participants). Although QuotaConfig allows researchers to specify enrollment minima or maxima, it does not provide power analyses or similar statistical evaluations. Like Cheatblocker, researchers can decide whether to inform potential participants they are ineligible automatically at the point of screening or to allow study staff to delay the quota check until the time of enrollment.

Researchers also select whether to set quotas for the entire sample or for smaller participant blocks. Blocked quotas ensure equal quota enforcement across study enrollment and may be particularly beneficial when a criterion involves a rare characteristic or otherwise difficult-to-recruit group of participants to avoid delaying all targeted recruitment until the end of the study. This feature also eliminates time as a confounding factor (e.g., avoid enrolling all men in 2025 and all women in 2026). Alternatively, setting quotas for the full sample ensures no participant is deemed ineligible because the quota has been reached in an earlier block when they would be eligible in a later block. Researchers should consider these contrasting advantages when using this tool.

QuotaConfig provides a framework for setting and calculating enrollment criteria that can be customized fully to a specific project’s needs. To date, this module has been installed 28 times across 8 institutions. Detailed instructions for using the QuotaConfig module are publicly available (https://github.com/MUSC-BMIC/redcap_quotas).

## Case studies

As case examples, we describe two projects that employed these tools within nicotine and tobacco cessation DCTs [23]. We selected examples from within our laboratory because we have necessary IRB approval among authors to permit full access to identifiable data from these projects. We discuss how we implemented the modules and our success with respect to detecting fraud and ensuring sample representation.

### COast project

We used Cheatblocker and QuotaConfig in a project conducted by Dahne and colleagues [24]. In this project, we developed and tested a remote carbon monoxide capture application (COast; integration of REDCap with the iCOQuit Smokerlyzer) with adults who smoke cigarettes for broad use in smoking cessation DCTs. We recruited online via Craigslist advertisements. Advertisements and the screening survey described the study as “paid,” but no payment details were disclosed until consenting to avoid impacting potential participants. Eligible, enrolled participants were compensated up to $166 (R21 CA241842). The study occurred fully remotely and relied on REDCap for all data collection and storage.

Initial study screening used an online survey that included a brief study description followed by screening questions. If participants were not eligible, they saw text thanking them for their interest, informing them they were not eligible, and providing free smoking cessation resources. All elements of the eligibility survey are customizable. We flagged records as potential duplicates and allowed study staff to make a final determination regarding study inclusion. We configured Cheatblocker to use the following criteria: identical first name *and* last name, *or* identical email, *or* identical phone number within a 6-month period.

Of the 4,844 completed screening surveys, Cheatblocker identified 1,497 duplicate records, comprising 30.90% of submitted surveys (Table 1). Duplicate records included 526 “original duplicates” (original record duplicated in subsequent record[s]) and 971 “duplicate entries” (subsequent records matching 1+ previous submissions). There was a range of 1–13 records within a given set of duplicates; in other words, some individuals submitted as many as 13 screening surveys. A breakdown of specific fields changed across duplicate records appears in Table 1. Almost 30% of duplicate entries were submitted on the same day as that participant’s previous submission, likely representing intentional fraud (Table 1). In contrast, the approximately 30% of duplicate entries separated by more than 1 month might represent genuine mistakes. Regardless of intention, Cheatblocker prevented enrolling the same person multiple times and preserved sample integrity.

Using the QuotaConfig module, we set the following enrollment minimums to ensure sample representation for the key, study-relevant characteristic of number of cigarettes smoked per day (cpd): 23 or more participants in each bin of 1–5 cpd, 6–10 cpd, 11–15 cpd, and 16+ cpd; and 36 or more participants who had quit within the last 30 days. Final sample (*N* = 143) characteristics demonstrate that we met these minimums: 1–5 cpd (*n* = 23; 16%), 6–10 cpd (*n* = 23; 16%), 11–15 cpd (*n* = 30; 21%), 16+ cpd (*n* = 31; 22%), and quit within the last 30 days (*n* = 36; 25%).

This project demonstrates successful implementation of Cheatblocker and QuotaConfig. DCTs often rely on remote recruitment (e.g., via online advertising that links to a web-based screening survey) to recruit quickly without the costly and time-consuming role of phone or in-person screening. The pace and reach of remote recruitment necessitate similarly automated processes to address fraud and sample representation. In this study alone, almost 5,000 online screening surveys were completed. Relying on study staff to review all screening submissions manually for fraud and to manage sampling quotas in real-time with a pool this large is not feasible. Cheatblocker and QuotaConfig allowed us to recruit a more representative sample with high data integrity.

Moreover, trusting our data allows us to conclude confidently that real participants who smoke found the COast app feasible and acceptable, and compliance was high for both daily or weekly CO monitoring [24]. These results are generalizable across individuals who smoke a wide range of cigarettes daily as well as individuals who have recently quit smoking. This project demonstrated feasibility and validity of remote capture of a critical biomarker for monitoring smoking cessation, which is valuable for DCTs that aim to mitigate the immense public health burden of cigarette smoking.

### VapeX project

We also used the Cheatblocker module in a recently completed DCT project conducted by Dahne and colleagues (clinicaltrials.gov ID number: NCT04951193) in which we developed, refined, and evaluated the mobile app “VapeX” to address depression symptoms and promote nicotine vaping cessation among older adolescents (ages 16–20). We recruited via social media advertisements (Facebook, Instagram, TikTok). Advertisements and the screening survey described the study as “paid,” but no payment details were disclosed until consenting to avoid impacting potential participants. Eligible, enrolled participants were compensated up to $190 (R41 DA053856). This study relied on REDCap for remote data collection and storage.

Study screening used an online survey that included a brief study description followed by screening questions. If participants were not eligible, they saw text thanking them for their interest, informing them they were not eligible, and providing free vaping cessation resources. All elements of the eligibility survey are customizable. We allowed study staff to make a final determination regarding study inclusion for records flagged as potential duplicates. We configured Cheatblocker to use the following criteria to flag potential duplicate records: identical first name *and* last name, *or* identical email, *or* identical cell phone number within a 6-month period.

Of the 2,845 completed screening surveys, Cheatblocker identified 713 duplicate records, comprising 25.06% of submitted surveys (Table 1). Duplicate records included 175 original duplicates and 538 duplicate entries. There was a range of 1–15 records within a given set of duplicates such that some individuals submitted as many as 15 screening surveys. Over 70% of duplicate entries were submitted on the same day as the previous submission, suggesting a high rate of intentional fraud (Table 1).

This project successfully implemented Cheatblocker among older adolescents. Younger individuals are more likely to volunteer for research, click through online screening links, and participate in online (vs. traditional) research due to reluctance to disclose sensitive information [5,20]. Additionally, adolescents may be more tech-savvy and subsequently more aware of how to fool potential fraud detection strategies. For example, Hardesty and colleagues [25] documented many fraud-related setbacks when conducting research with adolescents who vape. Indeed, there was a much higher proportion of same-day submissions in this case example compared to COast, which had an adult sample. These factors make it particularly important to demonstrate that Cheatblocker worked well with this population.

## Discussion

The purpose of this report was to address the translational science question of how we can improve data integrity within decentralized studies. DCT methods hold promise for mitigating bottlenecks in the research pipeline by increasing recruitment pace and reach and promoting broader, more inclusive participation. Alongside these benefits, however, concerns linger related to fraud and sampling bias, which may compromise data integrity.

We described two tools to address these threats to data integrity in DCTs. Cheatblocker helps to counter fraud by flagging potential duplicate records and denying these individuals study entry. QuotaConfig aims to mitigate sampling bias by monitoring designated quota characteristics throughout enrollment. These tools meet the need for proactive data governance – particularly “automated data validation checks” – required by the Good Clinical Practice framework ICH-E6(R3) [26], demonstrating the contemporary relevance and regulatory alignment of these proposed solutions.

These tools were designed with several key characteristics in mind to align with DCT methods and translational science goals. First, Cheatblocker and QuotaConfig are sufficiently automated to match the fast pace and high reach of remote recruitment. Automation ensures these tools reduce staff burden and streamline enrollment; however, options exist to rely on human verification of the smaller set of flagged records. Even this partially automated approach offers meaningful time saved; for example, in COast, staff only needed to review the approximately 31% (∼1500) of screening surveys identified as potential duplicates by Cheatblocker rather than almost 5000 total screening surveys. Second, these tools are generalizable across projects, investigators, and institutions and require minimal additional overhead. Third, Cheatblocker and QuotaConfig are customizable such that investigators can tailor the criteria in either module to be study-specific. Often generalizability and customizability work in opposition; striking this balance will promote broad uptake. Finally, these tools are integrated within REDCap, making them easily accessible to investigators at the thousands of institutions that use REDCap [21,22]. Instructions are publicly available. Thus, these tools are well-suited for wide-scale implementation within DCT research.

We demonstrated the successful use of Cheatblocker and QuotaConfig in two translational research case examples. These projects establish that these modules can address fraud and sampling bias to improve DCT research across adult and older adolescent populations [24]. Beyond these two examples, hundreds of research projects across dozens of institutions have used Cheatblocker and QuotaConfig, supporting their feasibility.

Fraud and sampling bias are known threats to data integrity in DCTs, necessitating proactive plans in DCT grants and protocols [11,12]. Indeed, the Good Clinical Practice ICH-E6(R3) framework stipulates “factors critical to the quality of the trial should be identified prospectively [26].” Fast-paced recruitment in DCTs means once recruiting begins, and there are already mass screening submissions, it is too late to put a plan in place. Hardesty and colleagues [25] chronicled the lessons they learned when trying to implement fraud detection strategies after screening in a longitudinal study of adults who vape. Other trials have faced similar difficulties, including suspending ongoing trials and excluding hundreds of data points [12,27]. The modules described herein are publicly available and integrated in the common research tool, REDCap, making them ideal, proactive solutions to cite in DCT grants and protocols.

Although we presented two case examples that used DCT methods, traditional clinical trials and other research could benefit from these same tools. The fast pace of recruiting without in-person verification makes concerns related to fraud and sampling bias particularly salient for DCTs, but the same concerns about data integrity exist for traditional trials. Implementing these tools could also support hybrid methodology (e.g., trials that employ online screening prior to in-person participation), which is increasingly common in clinical research [1]. These modules offer benefits across trial types that maximize data integrity while reducing research costs.

Despite the success of these tools thus far, combating threats to data integrity faces an ongoing major obstacle in that individuals who intentionally perpetrate fraud are often a step ahead of researchers who aim to deter it [5]. Thus, part of improving data integrity in DCT research must involve finding the next novel ways that participants may attempt to gain false study entry. Tools like IP address matching, platforms to detect virtual private networks (VPNs), CAPTCHA systems, improbable survey completion times, and consistency checks can help mitigate the threat of fraud [10,11,25]. Likely, combining multiple tools will be necessary to detect individuals perpetrating intentional fraud, bots, and other known and yet unknown sources of fraudulent data, not only at study screening but also during ongoing data collection [10,28]. Perhaps pilot trials for studies using DCT methods should screen for fraud and test various fraud detection strategies as part of demonstrating feasibility. This goal merits considerable thought and effort in future translational research.

Developing tools and resources that address roadblocks in the research pipeline is central to the mission of translational science. In a research world that increasingly relies on DCT methods and other digital tools, identifying possible threats to data integrity – including fraud and sampling bias – and developing associated solutions are critical. The tools that we have described to combat fraud and sampling bias are publicly available, easily implementable, customizable to individual projects, and sufficiently automated to match the pace of DCT research. Using these tools can increase confidence in the validity of our data and the representativeness of our samples.
